# Constructing a single market for pharmaceuticals in the EU: what’s the price?

**DOI:** 10.1017/S1744133125100236

**Published:** 2025-10-27

**Authors:** Tineke Kleinhout-Vliek, Susi Geiger, Rob Hagendijk, Eva Hilberg, Paul Martin, Katrina Perehudoff, Sarah Wadmann, Jakob Wested

**Affiliations:** 1 College of Business, University College Dublin, Belfield, Dublin 4, Ireland; 2 Faculty of Social and Behavioural Sciences, University of Amsterdam, Amsterdam, the Netherlands; 3 Department of Sociological Studies, University of Sheffield, Sheffield, UK; 4 Law Centre for Health and Life, University of Amsterdam, Amsterdam, the Netherlands; 5 VIVE, The Danish Center for Social Science Research, Copenhagen K, Denmark; 6 Faculty of Law, University of Copenhagen, Copenhagen S, Denmark

**Keywords:** European Union, pharmaceuticals, Health Technology Assessment, single market, pricing and reimbursement

## Abstract

The European Union (EU) is currently overhauling its pharmaceutical regulations, seeking to mature a single market for medicines as part of a ‘European Health Union’. We reflect on the interactions between regulations and markets in these reforms and investigate what this single market for medicines may mean in practice. We note how the proposed reforms aim to ensure equitable access to innovative treatments, yet at the same time, tie this access directly to regulatory exclusivities, limiting price competition. The reforms also do not seek full pricing transparency: prices will remain largely opaque and be set at the national levels rather than created through market exchange and open competition at the EU level. The envisioned single market for medicines thus remains a market that operates without direct reference to price − a situation not addressed head-on by the proposed reforms.

## Pharmaceutical reform and market construction in the European Union

‘Today we add another central pillar to our European Health Union. We are putting forward proposals to ensure that medicines reach patients everywhere in Europe, in a timely and equitable fashion. It is a reform which ensures that Europe remains attractive for business, and our pharmaceutical industry a global innovation powerhouse. Building a single market for medicines is a necessity both for our citizens and our companies’. − *Stella Kyriakides, Commissioner for Health and Food Safety, on presentation of the proposed reforms (26/04/2023)*

At this critical moment for EU public health (The Lancet Regional Health-Europe, 2024), we reflect on the interactions between regulations and markets in the ongoing negotiations of reforms to EU pharmaceutical legislation. Specifically, we focus on the proposed package for reforming the general EU pharmaceutical legislation, comprising a new Directive, a Regulation, and a Council Recommendation, and the adopted Health Technology Assessment Regulation (HTAR) currently coming into force. These together will form the basis of the EU’s pharmaceutical regulation for the next 10–15 years. Recent scholarship in economic sociology and science and technology studies has stressed that markets are the result of negotiations between actors in specific epistemic, institutional, and cultural contexts − there is nothing inevitable about either their organisation or the outcomes they achieve (Geiger *et al*., 2024). Instead, the establishment and functioning of markets result from specific organisational and institutional processes shaped by the technologies, practices, stakes, and norms of many interacting parties. These parties each bring their own goals and material interests into these practices of constructing markets, including in pharmaceutical markets (Geiger and Gross, 2018). In this paper, we problematise what this single market for medicines under construction means for European citizens’ access to (innovative) medicines and how this market is (to be) negotiated.

Concretely, the European Union pharmaceutical market has several features powerfully shaped by the way it is currently regulated: the dominance of large incumbent firms who extract significant profits, a high barrier to market entry, fragmented demand with purchasing delegated to national health care systems, limited and opaque price information, and, as a consequence, little competition and high prices in many therapeutic areas (Martin *et al*. unpublished) (Bourgeron and Geiger, 2022; Canoy and Versteegh, 2022). These structural market features show the enduring tensions between economic and health gains and how they tend to get resolved, one (innovative and often expensive) medicine at a time. Although these features seem to indicate that there are genuine limits to the room for manoeuvre and change, they also show the need for reform.

The current reforms we will examine comprise two parts. First, the HTAR (Regulation (EU) 2021/2282, amending Directive 2011/24/EU), which applies from 12 January 2025. HTA varies between member states and often limits the amount that will be reimbursed for pharmaceutical products. Moves towards a common European HTA framework have been intended to address this variance. This Regulation builds directly on this work and sets out a framework for strengthening EU Member State collaboration on the assessment of health technologies (an umbrella term for medicines and medical devices) (Desmet *et al*., 2024; Kanavos *et al*., 2019). Second, the proposed package for reforming the general EU pharmaceutical legislation comprising a new Directive (COM/2023/192), a Regulation (COM/2023/193), and a Council Recommendation (COM/2023/191) that will replace and amend important parts of the existing regulatory framework for the approval of medicines. These reforms result from long negotiation processes (see Table 1).

The objectives of the proposed reforms mirror a dual concern with equitable access to medicines and stimulation of pharmaceutical research and development (R&D) within the European Union. The currently proposed Directive and Regulation outline the requirements for medicine authorisation, monitoring, labelling, and protection for medicines authorised in the EU and its member states. The proposed Regulation sets additional rules concerning coordinated management of supplies and the governance of the European Medicines Agency (EMA). The Council Recommendation, finally, concerns combating antimicrobial resistance (AMR). The already-adopted HTAR comprises guidance for joint clinical assessments and scientific consultations and encourages joint identification of emerging health technologies (also known as horizon scanning) and voluntary cooperation between national HTA bodies.

The European Parliament adopted its position on the proposed Regulation and Directive on 10 April 2024, bringing the two proposals one step closer to reality. The detailed review and positioning by the Council (comprising the government leaders and relevant Ministers of the member states) was published in June 2025 and followed by the currently-ongoing ‘trilogue’ negotiations between Council, Commission, and Parliament, with agreement expected by the end of this year. The already-adopted HTAR applies to certain categories of pharmaceuticals as of January 2025. Thus, this paper is timely in analysing what we understand as the market construction aspects of these reforms. The reforms are embedded in a broader ambition, that of a ‘European Health Union’, which will chart the course of access to medicines in Europe for decades to come. We aim to shed light on a central issue that we assert should be urgently considered in the final negotiations leading up to the ratification of these reforms: the lack of transparent pricing mechanisms in this single EU market-to-be.

### A single market for medicines?

The EU pharmaceutical reforms aim to mature a single market for medicines (see Table 2) to solve the problem of differential access for European citizens. Significant differences in the availability of medicines exist across the EU. For instance, patients in Poland wait up to two years longer for the same innovative medicine than their neighbours in Germany (EFPIA, 2022), and many medicines do not get marketed in every member state (Németh *et al*., 2022). While the problem of inequitable access across the EU is given as the primary reason for the proposed reforms, underlying and interconnected problems identified by the Commission include: the market selection decisions of pharmaceutical companies, diverging regulatory procedures, supply-chain issues, and the high prices and lack of transparency in pharmaceutical pricing (European Commission, 2020a, 2022b, 2023; European Union, 2024).

Differential access to pharmaceuticals is the result of a specific combination of current EU and national regulations and practices. While most other goods are bought directly by end users, prescription medicines are either purchased or subsidised by national health care systems or their designated representatives (such as insurers or hospitals) and then sold or administered to patients through national health care systems. HTA authorities and economic evaluation bodies recommend whether a medicinal product should be bought at the price set by the manufacturers or whether limits should be set, generally highly contingent on the setup and priorities of national health care systems. The use of (external) reference pricing is contested and any evidence concerning its effects is considered ‘weak’ (Kanavos *et al*., 2020), apart, perhaps, from causing entry delays and higher prices in lower-income EU countries (Elek *et al*., 2017; Maini and Pammolli, 2023). Indeed, these dynamics render especially smaller and lower-income European countries worse off in terms of access to medicines and health outcomes more generally (EFPIA, 2022; Németh *et al*., 2022).

In the EU, policies with respect to medical care and most of public health are the prerogative of member states and their governments, including the provision of pharmaceuticals, their pricing and reimbursement. Yet the marketing authorisation of new medicines has been centralised through the EMA since 1995. The EU only has partial responsibility for public health issues: well-known areas include cross-border health care and medicine quality. Currently, the EU is also not to interfere with the economic rights of companies and persons to set the price as they see fit when selling products, assets or services that are legally their property. Notably, the current regulatory proposals do not affect national patent law. Exceptions to the free pricing principle include cases of abuse of dominant market position (Veraldi, 2024). This division of sovereign competencies of states and entrepreneurial freedoms has long restricted the regulatory options for EU legislators to create a single market for medicines.

The COVID-19 pandemic, however, critically underlined the need for EU-level collective action and entailed such collaboration in the form of collective vaccine price negotiations and purchases. To frame future collaboration, European Commission President Ursula von der Leyen first mooted the idea of a European Health Union in her 2020 State of the Union. So far, this European Health Union primarily entails responding better to cross-border threats and increased collaboration in terms of data exchange (Council of the EU, 2024; McKee and de Ruijter, 2024). The pandemic hit at a time of increasing collaboration between member states’ HTA bodies in the face of rising medicine prices (Ruether *et al*., 2022). Crucially, then, the proposed reforms build on and interact with existing regulative, legal, and policy frameworks, with the overall aim to advance the European Health Union, of which a mature and well-developed single market is to act as a ‘central pillar’ (see quote above).

### The proposed reforms

So, how does the Commission propose to work towards this single market for pharmaceuticals given the boundaries and constitutive principles of the EU (mentioned in Table 2)? The proposals in the recently-presented general pharmaceutical legislation seek to achieve this by providing specific incentives to pharmaceutical companies. To do so, this part of the reforms include a range of new and changed regulations and exemptions (for several of these points, more information may be found in Appendix 1). First, when it comes to improving regulatory pathways, the reforms propose a) offering tailored scientific support to developers of innovative products years before they apply for marketing authorisation, b) increasing the use of ‘rolling reviews’ (ie, a phased regulatory review of data as they become available), c) introducing ‘regulatory sandboxes’ to test new regulatory approaches, and d) facilitating the use of ‘real-world evidence’ and digital technologies, including artificial intelligence. Second, the proposals contain policies to address several situations considered problematic, such as institutionalising temporary marketing authorisations or compulsory intellectual property licenses for public health emergencies, strengthening possibilities for pharmacist and hospital preparations or compounding for (temporary) unavailability, and extending the scope of the ‘hospital exemption pathway’ (Coppens *et al*., 2020). Third, the proposals expand the scope of the so-called ‘Bolar exemption’, which allows generic and biosimilar manufacturers to use patent rights underlying an innovative product when preparing regulatory filing for their version. Fourth, to address the environmental implications of pharmaceutical research, production, distribution, and use, the reform seeks to strengthen the requirement for companies to conduct environmental risk assessment (ERA) of medicines. Fifth, transparency on financial support received from public authorities and publicly funded bodies is mandated for newly-marketed medicines, to be published on company websites and the database of medicinal products for human use authorised in the Union. Finally, and most critically for our argument, the proposal introduces a ‘modulated system’ of incentives.

The ‘modulated system’ of incentives is a central part of the proposed reforms. We foreground this as it is highly significant in light of the drive towards a single market for medicines, both in terms of the public debate it sparks and when comparing the current iteration with the initial Commission proposals. This modulated system uses regulatory data protection periods as the primary tool to incentivise pharmaceutical innovation. Regulatory data protection prevents generic or biosimilar companies from referring to the originator’s R&D and clinical trial data to support the former’s own marketing authorisation applications. Commission and Parliament agree to reduce the standard regulatory data protection period, currently set at eight years. However, they differ as to whether the standard protection should last for six or seven and a half years − and the Council’s position now sets it back at eight years. In the modulated system, companies can earn additional periods of regulatory protection in cases of perceived market failure, including rare diseases and conditions where no competitor product has been marketed yet, representing (high) unmet need. Further protection can be earned by conducting clinical trials with an active comparator, conducting (part of) the research in the EU or with EU-based partners, and identifying additional medicine uses for other indications (also known as repurposing). Some of these incentives, including the rare disease category, already received extended protection under the current system; others are new public health goals to be addressed in this way, such as AMR and vaccine accessibility.

The underlying assumption of the above-mentioned incentives concerns the effects of lower competition on increased revenues that would be re-invested in pharmaceutical R&D by the companies. While some argue that this will benefit patients’ (future) access to medicines, others remain skeptical (Canoy and Versteegh, 2022; Geiger, 2025). Instead, the situation concerning drugs for rare diseases shows that an increase in competition due to more marketed products may also act as an industry deterrent in terms of R&D investments. In fact, these incentives may have little overall impact on corporate behaviour, especially in cases of high unmet medical need (Martin *et al*., unpublished; European Commission, 2020c). Still, if these proposals are adopted, access to new and often highly-priced treatments will be tied to extended market exclusivities − limiting price competition in the name of access.

### Lack of transparent pricing mechanisms

Ample works in economic sociology have established the central importance of price as a coordination device (Muniesa *et al*., 2007). Yet, analysing the reforms and particularly the incentives directed at alleviating market failures in medicines markets, it is evident that these reforms ‘miss’ a pricing component, so to speak. Even though the European Commission’s press release accompanying the proposed reform of the general pharmaceutical legislation states that ‘the reform addresses the entire lifecycle of medicines’ (European Commission, 2023), pricing is not among them. In the current proposals, pricing and reimbursement issues remain the sovereign responsibility of the member states (see 6 TEU, 168 TFEU, 35 Charter EU). As the EU has historically not concerned itself with transparency of R&D costs and (net) prices of medicinal products, individual member states have dealt with transparency regarding pricing and reimbursement in different ways. The Transparency Directive of 1989 created binding rules for member states negotiators to disclose their timelines − not paid prices (Geiger and Bourgeron, 2023). Moreover, while pharmaceutical companies have been pushed toward greater price transparency by the resolution initiated by Italy and passed by the World Health Assembly in May 2019 (Perehudoff, 2022), the EU is not involved in the adoption of this rule, as member states hold this responsibility at the national level. The continent has seen the institutionalisation of reference pricing mechanisms, with prices negotiated elsewhere used as benchmarks, nominally tying prices together across the EU (Doganova and Rabeharisoa, 2024; Kjellberg *et al*., 2023; Rémuzat *et al*., 2015). Over the past years, member states have started collaborations such as the Joint Nordic HTA Bodies (JNHB), BeNeLuxA, and Valletta initiatives to negotiate prices together; the first collectively negotiated prices are now a fact (Pisarczyk *et al*., 2018; Vogler *et al*., 2021). Even more recently, during the COVID-19 pandemic, the EU Commission’s lack of price transparency concerning the jointly procured vaccines is widely understood to indicate a lack of commitment in this regard, as transparency about (net) purchase prices would have been possible without infringing on member state competences (Boulet *et al*., 2021). Indeed, while explicitly seeking to construct a single market, the proposed general pharmaceutical legislation reforms only address pricing indirectly: a mandatory reporting of public R&D funding received by companies is to provide national procurers a better stance in price negotiations with pharmaceutical companies.

The HTAR, building on existing price negotiation collaborations, does elaborate on the issue of pricing. The HTAR is of great significance here, as in most EU countries, HTA bodies make recommendations on the use of new, expensive medicines in national healthcare systems based on cost–benefit analysis, thereby framing any subsequent price negotiations, as outlined above. This Regulation seeks to harmonise the requirements for documentation of relative clinical effectiveness of intervention. The HTAR comes as a promising policy move. It is welcomed from many sides, as more collaboration on HTA simultaneously promises greater speed, less bureaucracy, and more negotiation power vis-à-vis industry on behalf of a greater number of citizens − at least in countries where procurement is also centralised. At the same time, this move is likely to decontextualise decisions as there is less room for differences between member states (Kleinhout-Vliek *et al*., 2020). It may also depoliticise decisions, threatening their democratic legitimacy with decision-makers at a greater distance from those the decisions concern (Löblová, 2021). Whether transnational HTA bureaucracy change will advance pricing principles and systems is debatable, especially because of interacting issues, such as diverging models of health care funding, contracting and delivery, and co-payment across member states (European Observatory on Health Systems and Policies, 2024).

In sum, while streamlining approval procedures seeks to ease the way for international pharmaceutical companies, current national price-setting practices, often conducted outside public accountability by reference to trade secrecy, are not fundamentally challenged by these reforms. While European reference pricing has held promises to increase price transparency across Europe, this famously has only led to pharmaceutical pricing becoming ever more dependent on secret rebates; published list prices have veered further away from the ‘real’ prices paid by national governments and insurers (Doganova and Rabeharisoa, 2024; Kjellberg *et al*., 2023). The proposed reforms do not tackle these issues. High prices threaten the sustainability of member states’ health care systems as they affect patient access through delayed market entry, high co-payments, negative reimbursement decisions, and rationing. These issues are particularly pressing in lower-income European countries, but occur increasingly in higher-income countries too (Wadmann *et al*., 2023). Still, there are no proposals to require full price transparency.

Further, no effort is made to strengthen or build on pre-existing efforts in this area, prominently including the Oslo Medicines Initiative (Larsen *et al*., 2022), the meetings of EU Competent Health Authorities to compare notes about pricing and negotiations with industry (known as the NACPR meetings), and price-negotiating collaborations like BeNeLuxA. Critically, this limits price competition and leaves health care systems vulnerable to exploitation by commercial entities (Geiger and Bourgeron, 2023). This also foregrounds another systemic issue: the pervasive policy focus on ‘affordability’ rather than on the prices actually paid. This difference is not purely semantic but implies a shift of the burden of proof from the industry to individual national health services (or insurance plans) to show that there may be long-term issues concerning the affordability of a given medicinal product. While evidence might become available over time on whether a payer has overpaid for a medication (Jommi *et al*., 2023), the price of many high-end therapies remains high and potentially unaffordable for some member states. Pertinent questions include: How will information about the public funding received affect industry pricing practices? What might the effect on competition be? Will there be political will to encourage discussions and disclosures of real paid prices?

### Outlook

In EU member states, market mechanisms can be harnessed to serve social goals in the distribution of goods and services through appropriate regulation. While problems of medicine access have been longstanding, the current reforms seek to simultaneously establish a single market and a social and political union: the European Health Union. We argue that the proposed pharmaceutical reforms do not fully address the central problem of accessibility of medicines in the European Union head-on: the pricing of (innovative) medicines. Instead, the Commission seeks to achieve a single market without claiming any authority over pricing mechanisms, nor does it seek to increase the transparency of national pricing processes, disabling meaningful price comparisons. This situation is only moderately alleviated by increased transparency about the contribution of public funding to the development of a new drug. Therefore, differential access is likely to continue as long as price-setting negotiations take place behind closed doors, leaving the prospect of a genuine ‘European Health Union’ a long way off.

We contend that the time is ripe for the EU’s lawmakers and public authorities, including the European Commission and member states’ competent authorities, to assume greater responsibility in constructing a single EU market for medicines in a way that enables price transparency and new ways of assessing the economic and social value of a medicine. The importance of such a change is also underscored by the recent case before an Amsterdam court questioning whether the sale price of Humira in the Netherlands yielded excessive profits, in *Stichting Farma ter Verantwoording v. Abbvie* (Rechtbank Amsterdam, 2025). To do so consistently and across the board requires a different approach to pricing, especially in areas of contention and perceived market failure, such as rare diseases, AMR, and, to some extent, drug repurposing (Anderson *et al*., 2023; de Visser *et al*., 2024; Mazzucato and Roy, 2019; Scholte *et al*., 2025). ‘Full transparency’ clearly is an aspirational project that would require sustained, long-term political will and action to achieve for two reasons. First, ‘transparency’ has many facets, including clinical trial data and cost transparency (which companies would provide) and net paid price transparency (to be provided by payers) (Geiger and Bourgeron, 2023). Achieving full transparency of all these facets will likely require a step-wise approach by governments and the EU. Such steps are already being taken (Medicines, Law & Policy, 2019), for example, through the EU requirement to declare R&D costs and revenues of a product as one avenue through which a company may obtain an orphan drug designation. However, ample room remains for additional measures. Second, as outlined above, the EU does not have the legal power to intervene in national price-setting practices, including regarding what price information is disclosed to member state authorities. However, there are other powers that the EU can leverage to support transparency, such as its powers of coordination in the field of public health.

All practical steps towards increasing any form of transparency (especially those provided by companies) remain helpful in regaining public control and oversight over public spending and building the envisioned single market. In particular, we welcome EU efforts to remedy high prices and decrease inequity of access within the limits of its legal powers through the EU pharmaceutical review. In line with the HTAR, we would encourage even greater collaboration on pharmaceutical pricing, strongly building on and promoting initiatives like the Oslo Medicines Initiative, the International Horizon Scanning Initiative, and the JNHB, BeNeLuxA, and Valletta initiatives mentioned earlier (Larsen *et al*., 2022; Pisarczyk *et al*., 2018). Concrete steps could include mandating a declaration of all prior funding received (not just that of public institutions) as well as on all R&D costs and expected revenues to demonstrate the need for a certain number of years’ exclusive marketing rights (‘sufficiency principle’). Additional options may involve developing and using alternative pricing models (Godman *et al*., 2021; Manders *et al*., 2024). These include ‘cost plus’, which hinges strongly on development and production costs instead of (projected) added therapeutic value, and comes with its own challenges for determining these costs in a transparent, accountable manner (Johansen *et al*., 2025). A second example is multi-indication pricing, which is strongly value-based, but may be used as a cost-containment strategy as part of outcome-based pricing agreements (Mestre-Ferrandiz *et al*., 2018). Another option would be introducing the possibility of waiving the transferable data exclusivity voucher when the reference price on the EU market is over a certain percentage of the declared R&D costs, or, a step further, differential, GDP-adjusted pricing across the EU, which has long been explored by health economists but has yet to be implemented (Danzon and Towse, 2003; Kaló *et al*., 2013; Towse *et al*., 2015). Finally, we suggest promoting exchange between member states’ HTA agencies on societal value arguments concerning reimbursement, which may yield additional reasons for making negative and positive decisions alike societally robust (Kleinhout-Vliek *et al*., 2021).

Finally, a European Health Union that meets the needs of all EU citizens equitably may require governments at all levels to pro-actively engage in the field in a ‘mission-oriented’ manner, for instance, through setting up a union-wide public health authority that seeks to incentivise, and perhaps execute, R&D for medicines where the current system is not delivering (Chatelain and Ioset, 2011; Douglas *et al*., 2022; Kleinhout-Vliek *et al*., 2024; Public Pharma for Europe, 2024). A hopeful first indication is the waning of some member states’ ‘neoliberal belief in the merits of competition in health care’ (Maarse and Jeurissen, 2024). A union-wide public health authority would seek to direct industrial innovation by providing a much greater range of policy instruments and incentives and tighter regulation to avoid gaming and abuse of the regulatory system − and to build affordability of medicines and medical technologies into their R&D cycles (Suleman *et al*., 2020). This regulation could be the start of a new ‘social contract’ with the European public and pharmaceutical industry to ensure the sustainability of the sector and access to medicines for EU citizens and further afield (Lemmens *et al*. 2022).

## Supplementary Material

To view supplementary material for this article, please visit https://doi.org/10.1017/S1744133125100236.

Supplementary Material

Appendix

## Figures and Tables

**Table 1:** Legislative timelines

Month year	Adoption of EU Regulation on HTA	Revision of general EU pharmaceutical legislation
October 2016	Launch of online public consultation on the future of EU collaboration on HTA (European Commission, 2016)	
January 2018	Proposal for a Regulation on HTA formally adopted by the Commission (European Commission, 2018)	
February 2019	European Parliament adopted its position on the Regulation on HTA (European Commission, 2024)	
December 2019	Pharmaceuticals were a main concern in President of the European Commission Ursula von der Leyen’s mission letter to Commissioner for Health and Food Safety Stella Kyriakides (Von der Leyen, 2019)	
November 2020		European Commission adopted the Pharmaceutical Strategy for Europe, of which reforming the existing EU pharmaceutical legislation package is ‘the main flagship action’ (COM/2020/761) (European Commission, 2020b)
March 2021		Commission published a Combined Evaluation Roadmap and Inception Impact Assessment for the revisions
November 2021	Council of the EU adopted its position on the new Regulation on HTA (European Commission, 2024)	
December 2021	Final adoption of the new Regulation on HTA (European Commission, 2024)	
May 2022	Conclusion of the citizen-led Conference on the Future of Europe, which urged a revision of the Treaty on the Functioning of the European Union to recognise health and health care as competencies shared between the EU and member states (European Commission, 2022b)	
April 2023		European Commission published its proposals for revising the EU general pharmaceuticals legislation in the form of a Directive, Regulation, and Communication
April 2024		European Parliament adopted amendments to the Directive and Regulation by an overwhelming majority (European Parliament, 2024)
June 2025		European Council agreed on its position; trilogue negotiations between Council, Commission, and Parliament commenced (Council of the EU, 2025)

**Table 2:** EU single market in pharmaceuticals

The EU first introduced its single market policies in the Single European Act of 1986, intending to abolish all non-tariff obstacles to trade between its member states. This measure addressed various issues that had previously prevented ‘free trade’ between states and aimed to bring about job growth and increased trade between individual countries. The envisaged EU single market would operate on the basis of ‘four freedoms’: free movement of goods, free movement of capital, freedom to establish and provide services, and free movement of people. Sector by sector, barriers interfering with the four freedoms were identified and harmonisation measures and agreements were negotiated to advance the realisation of the single market goal. Pharmaceutical products as goods fall under the free movement norm: if it has been placed on the market in one member state, it can be sold on any other member state’s market by default. Parallel importing, though associated with medicine shortages in member states where prices are lower (Zaprutko *et al.,* 2020), is explicitly allowed under a parallel import marketing authorisation, which may be granted if the product has received a marketing authorisation in the member state of origin and the product is sufficiently similar to one already authorised in the member state of destination. This does not apply to products with a ‘community’ marketing authorisation issued by the European Commission. Any further limitations of this free movement by member states (or private actors) must be justified either based on the Treaty on the Functioning of the EU (TFEU) article 36 or case law of the EU Court of Justice (Ref. Cassis de Dijon 120/78). Justifications of relevance to medicinal products include public health and the protection of industrial and commercial property rights, which encompass intellectual property rights (IPR). This means that national authorities can impose additional requirements to placing a medicinal product on the national market even though the product has already been placed legally on the market within the EU to protect public health or IPR. National authorities may require information such as the summary of product characteristics (SMPC) translated to the national language, and a valid patent right in one member state may hinder the market entry of a generic producer. However, it can be marketed in states where no patent right is in force.

## Data Availability

Data availability is not applicable to this article as no new data were created or analysed in this study.
