# Supplementary material for: Constructing a single market for pharmaceuticals in the EU: what’s the price?
Source: Health Econ Policy Law. Author manuscript; Available in PMC 2026 Jan 7. (PMC7618563; doi:10.1017/S1744133125100236)
Supplement: Supplementary Material [file EMS211601-supplement-Supplementary_Material.docx]

Appendix 1 – Legislative Background

**Bolar exemption**: Specifically, increased generic competition is being encouraged as the main way to reduce prices by making regulatory data available earlier, but there are bottlenecks in the capacity of existing generic manufacturing as illustrated by recent supply problems, and most innovative medicines still enjoy comprehensive patent protection. The slow progress in introducing biosimilars also highlights some of the regulatory and technical difficulties in increasing competition. The Bolar exemption allows generic manufacturers to use patent-protected inventions for the purpose of preparing and applying for a marketing application for a generic or biosimilar product. The proposal clarifies a broader scope of this exemption to patent protection.

**Transferable data exclusivity voucher**: The voucher provides one extra year of data exclusivity that may be sold on. This decouples the financial incentive from the innovation, which is vital in the case of antimicrobials, as limiting the use of a new antimicrobial is crucial to its added value.

**Modulated system of incentives**: Patent protection and regulatory data protection are different systems of protection and the reform proposal does not concern the system of patent protection. Data protection implies that generic manufacturers cannot refer to the data produced by the license holder of an innovative medicines product to obtain market authorisation.

**Regulatory data protection period**: In the initial Commission proposals, the basic data protection period was reduced from eight to six years, with additional years of protection conditional on certain targets. This shows that Parliament seeks to largely uphold the EU’s regulatory protection, which is significantly longer than in most other high-income countries, where six years of data exclusivity is the standard. However, the possibility to extend the protection sustains the level of EU data exclusivity at a high level.

**Modulated market exclusivity for orphan drugs**: We see an additional modulation of market exclusivity for medicines for rare diseases, though with different features. Products seeking marketing authorisation based on well-established use only get a market exclusivity of five years. The remaining orphan drugs must address a high unmet need to receive a basic market exclusivity of 10 years, and if not, only nine years, with a maximum of thirteen years (now ten years) by meeting further requirements.

**Repurposing or repositioning**: Repurposing, in which an existing approved medicine is used to treat a new disease indication, has long been a critical modality of orphan drug development, providing a relatively cheap and expedient strategy to develop new treatments (Wested and Liddicoat, 2021). The proposed reforms suggest a maximum of four additional years of market exclusivity here (Scholte *et al.*, 2025). However, under the current regime, repurposed drugs have often received a separate designation and thus full 10 years of market exclusivity.

**Mandatory reporting of public R&D funding**: Half a year of additional regulatory data protection can be obtained by having an active comparator in clinical trials. This can also be interpreted as a means to support the assessment conducted by HTA committees, providing them with data on the relative efficiency of an intervention at an earlier stage.

**Concurrent EU launch**: An additional period of regulatory protection was initially proposed for a concurrent launch in all EU member states. This requirement of concurrent product launch across all member states tied a bonus year of market exclusivity in preamble 103 of the proposed regulation to “a Union market launch” – the required parameters of which remained entirely unclear in the text. First reactions from stakeholders in different corners soon raised questions about the feasibility of such a launch, as they will in practice differ based on member states’ HTA processes, and the dropping of this requirement has been met with relief from industry, particularly. This criterion has been replaced by a requirement to apply for ‘pricing and reimbursement’ when asked to do so by the competent authority of a certain member state, but with a number of exemptions, to be listed by the EMA and the Commission, who will also resolve disputes. The Commission will also develop ways to measure how the EU health care landscape is evolving in terms of access and facilitate the exchange of best practices between member states.
